# Cellular dynamics during early barley pollen embryogenesis revealed by time-lapse imaging

**DOI:** 10.3389/fpls.2014.00675

**Published:** 2014-12-08

**Authors:** Diaa Eldin S. Daghma, Goetz Hensel, Twan Rutten, Michael Melzer, Jochen Kumlehn

**Affiliations:** ^1^Department of Physiology and Cell Biology, Leibniz Institute of Plant Genetics and Crop Plant ResearchGatersleben, Germany; ^2^Department of National Gene Bank and Genetic Resources, Agriculture Research CenterGiza, Egypt

**Keywords:** barley, pollen, embryogenesis, live-cell, imaging

## Abstract

Plants display a remarkable capacity for cellular totipotency. An intriguing and useful example is that immature pollen cultured *in vitro* can pass through embryogenic development to form haploid or doubled haploid plants. However, a lack of understanding the initial mechanisms of pollen embryogenesis hampers the improvement and more effective and widespread employment of haploid technology in plant research and breeding. To investigate the cellular dynamics during the onset of pollen embryogenesis, we used time-lapse imaging along with transgenic barley expressing nuclear localized Green Fluorescent Protein. The results enabled us to identify nine distinct embryogenic and non-embryogenic types of pollen response to the culture conditions. Cell proliferation in embryogenic pollen normally started *via* a first symmetric mitosis (54.3% of pollen observed) and only rarely did so *via* asymmetric pollen mitosis I (4.3% of pollen observed). In the latter case, proliferation generally originated from the vegetative-like cell, albeit the division of the generative-like cell was observed in few types of pollen. Under the culture conditions used, fusion of cell nuclei was the only mechanism of genome duplication observed.

## Introduction

The life cycle of higher plants involves the alteration of sporophytic and gametophytic generations. Whereas the sporophyte constitutes the plant in its apparent form, the female and male gametophytes are reduced in size and have become depending on the sporophyte.

Under natural conditions, spontaneous formation of haploid embryos and plants in angiosperms can arise from female gametophytic cells but not from male gametophytic cells (McKone and Halpern, 2003). However, male gametophytes (i.e., immature pollen) cultivated *in vitro*, can be induced to become embryogenic and form sporophyte-like haploid embryos and plants. Through spontaneous or artificially triggered genome duplication, doubled haploids can arise that themselves are genuine sporophytes.

The value of haploid technology in plant research and breeding lies in the fact that the founder cells of doubled haploids are products of meiosis, and resultant plants constitute pools of diverse recombinant, yet genetically fixed individuals. Their recombinant genome is fixed through homozygosity of the doubled haploid plants produced. The employment of haploid technology has become widely used in breeding programs of many crop species (Germanà, 2011).

Doubled haploids are also widely used in genetic studies, such as QTL, gene mapping and marker-trait association (Murovec and Bohanec, 2012). Genetic transformation of embryogenic pollen allows for the production of instantly true-breeding transgenic plants (Kumlehn et al., 2006; Eudes and Chugh, 2008; Chauhan and Khurana, 2011). A novel approach for haploid induction has recently been developed by the genetic engineering of the centromeric region (Ravi and Chan, 2010).

Although distinct pathways of pollen embryogenesis have been proposed (Sunderland and Evans, 1980; Hu and Kasha, 1999), recent evidence hints at the presence of multiple pathways within one culture (Hu and Kasha, 1999; Kasha et al., 2001). Maraschin et al. (2005), using time-lapse studies, identified three such pathways within immature barley pollen cultures.

Spontaneous genome doubling can result in completely fertile doubled haploids. In barley and wheat, spontaneous doubling frequencies between 18 and 85% have been observed, (Jähne and Lörz, 1995; Hu and Kasha, 1999). Several mechanisms for plant genome doubling have been proposed: (i) endoreduplication, (ii) nuclear fusion, (iii) endomitosis, and (iv) c-mitosis (Jensen, 1974; d'Amato, 1989; González-Melendi et al., 2005; Kasha, 2005; Seguí-Simarro and Nuez, 2008). However, none of the proposed genome doubling mechanisms has ever been observed in living cells.

Despite the great value of pollen embryogenesis, very little is known about the underlying cellular mechanisms. In vacuolated immature pollen, embryogenesis can be induced by various treatments both *in vivo* and *in vitro* (Touraev et al., 1997). Due to the high amenability to pollen embryogenesis, barley has become a model species to study this phenomenon in temperate cereal crop species (Sunderland et al., 1974; Kasha, 2007).

This present paper is the first to give a full account of the initial cellular dynamics in the pollen embryogenesis process until the formation of growing multicellular structures. The approach relies on multi-dimensional (4D) live-cell imaging of transgenic pollen expressing the *Green Fluorescence Protein* (*GFP*) gene with nuclear localization signal using a temporal resolution of 3 min.

## Results

### Generation of transgenic barley expressing SV40-NLS:GFP

**Figure 1:**
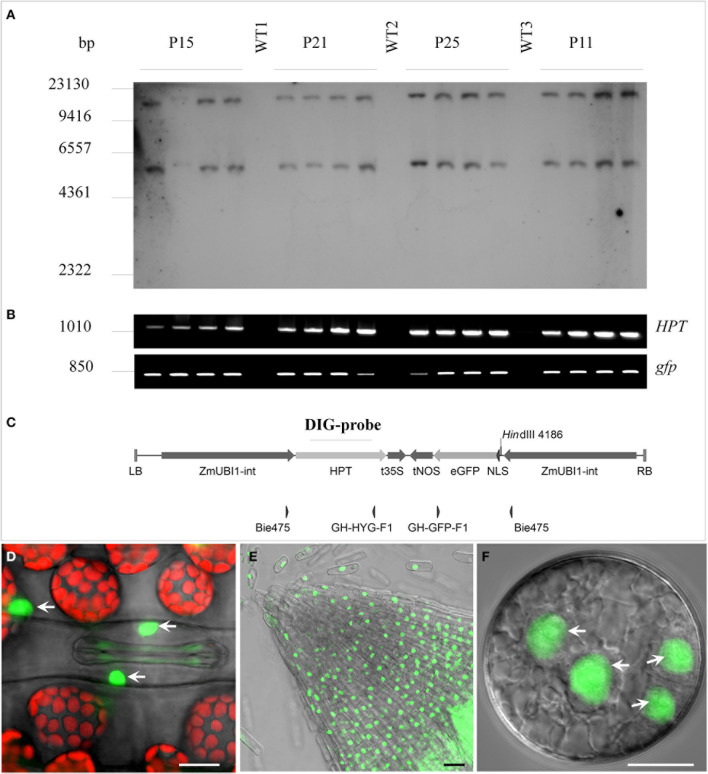
**Characterization of the doubled haploid *SV40-NLS:GFP* transgenic barley line displaying nucleus-specific accumulation of GFP**. Randomly chosen T_2_-siblings (four per family) derived from four T_1_-plants (P11, P15, P21, P25) were analyzed for genomic T-DNA integration and transgene zygosity. **(A)** DNA gel blot analysis of *Hin*dIII-digested genomic DNA hybridized with an *HPT*-specific probe. The two bands seen per lane indicate genomic integration of two T-DNA copies. **(B)** PCR analysis with primer pairs specific for the *HPT* (upper band) and the *GFP* (lower band) genes. **(C)** Map of T-DNA with primer pairs and hybridization probe positions indicated. WT1, 2 and 3, wild type individuals of cv. “Igri”; LB, left border; ZmUBI1-int, maize *UBIQUITIN1* promoter with first intron; HPT, hygromycin B phosphotransferase protein coding region; t35S, *CaMV 35S* gene terminator; tNOS, *NOPALINE SYNTHASE* gene terminator; eGFP, synthetic S65T green fluorescent protein coding region; NLS, SV40 Simian virus 40 nuclear localization signal; RB, right border. **(D)** Leaf tissue with GFP in the nuclei of guard and other epidermis cells. Chlorophyll autofluorescence shown in red. **(E)** GFP accumulation in the nuclei of a root tip. **(F)** GFP accumulation in nuclei of immature pollen after the second embryogenic pollen mitosis. Bar = 30 μm.

A total of 71 primary transgenic (T_0_) plants were generated by co-culture of embryogenic pollen with *Agrobacterium* strain LBA4404/pSB1. This strain harbored a *GFP* gene fused to the Simian virus SV40 nuclear localization signal under the control of the maize *UBIQUITIN1* promoter with first intron (Figure 1C). Ploidy and presence of the selectable marker gene *HPT* were checked by flow cytometry and PCR, respectively. Most regenerants were haploid (43.7%) or diploid (47.9%), while the remaining ones were tetra- (5.6%) or mixoploid (2.8%). Five spontaneously doubled haploid and 20 colchicine-treated plants were grown to maturity. Three colchicine-treated haploids did not set grain. The remaining 22 fertile doubled haploids contained the *HPT* selectable marker gene. Confocal Laser Scanning Microscopy (CLSM) analysis showed only a single line to accumulate appreciable amounts of GFP in its nuclei. This line was used to produce embryogenic pollen cultures from which 27 T_1_ regenerants were generated including 7 tetraploid, 5 triploid, and 15 diploid plants. Sexual progeny of 7 randomly selected diploid T_1_-lines were grown. To confirm integration of *HPT* and *NLS:GFP*, four T_2_-families were selected at random for DNA gel blot analysis (Figure 1A) and PCR (Figure 1B). Primer pairs and specific probe were selected to cover most functional parts of the T-DNA (Figure 1C). All plants contained both *HPT* and *NLS:GFP* (Figure 1B) and showed an identical T-DNA integration pattern (Figure 1A), confirming that the doubled haploid T_1_-lines were homozygous for the transgene. Nuclear localization of GFP in T_0_ transgenic plants was confirmed by CLSM (Figures 1D–F).

### Live-cell imaging of cultured pollen over time

In eight separate experiments, the development of a total of 71 immature pollen was followed over a time span of up to 15 days. Of these pollen, 70 were (uni-nucleate) microspores and a single one bi-cellular. With a diameter of about 40 μm, the bi-cellular pollen was distinctly larger than the microspores, which were about 30 μm. Over time, the bi-nucleate pollen increased in size without showing any mitotic activity and started to accumulate starch (Supplementary Figures 1C,D) before dying on the second day of culture (Supplementary Figures 1E,F; see Supplementary movie 1). The small generative-like nucleus of this bi-cellular pollen remained spherical (Supplementary Figures 1A–D; see Supplementary movie 1). The remaining 70 immature pollen that were at the late microspore stage at the onset of observation showed various developmental patterns with regards to pollen mitosis I and to their final fate, so that nine different developmental pathways could be discerned (Table 1). The majority of pollen (61.1%; types I, II, and III) started the development with a symmetrical cell division, whereas types IV, V, and VI had in common to undergo asymmetric pollen mitosis I. In contrast to types I–VI, no mitotic activity was observed in types VII–IX.

**Table 1:** **Types of pollen development as observed in live-cell imaging experiments during the initial 2 weeks of culture under conditions supportive for pollen embryogenesis**.

**Developmental type**		**No. of pollen**	**% of total pollen**
**SYMMETRIC MITOSIS I**			
Embryogenic	I	38	54.3
Bi-nucleate, starchy, collapsed	II	2	2.9
Bi-nucleate, starchy, survived	III	3	4.3
**ASYMMETRIC MITOSIS I**			
Embryogenic	IV	3	4.3
Division of generative cell, no starch, survived	V	2	2.9
Bi-cellular, starchy, collapsed	VI	5	7.1
**FAILED MITOSIS I**			
Micronuclei formation	VII	1	1.4
Pollen expansion	VIII	2	2.8
No development	IX	14	20.0

**Figure 2:**
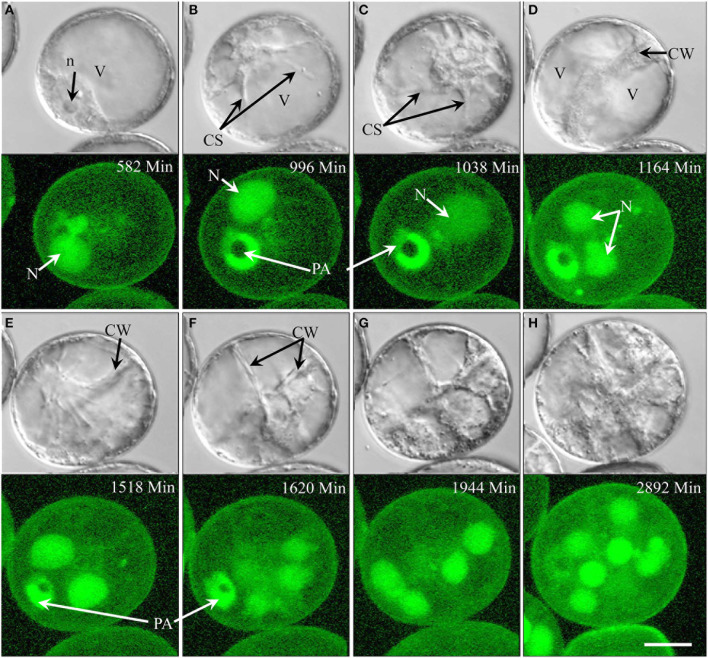
**Time-lapse of type I development (embryogenic pollen) shown by synchronously acquired DIC and fluorescence images. (A)** Uni-nucleate pollen (microspore) with nucleus close to pollen aperture. **(B,C)** The nucleus has migrated away from the pollen periphery and cytoplasmic strands are formed. The blurred fluorescence signal indicates the break-down of the nuclear envelope prior to mitosis. **(D)** Newly formed cell wall (DIC) and a pair of daughter nuclei (GFP) after mitosis. **(E)** Appearance of cytoplasmic strands indicating imminent second mitosis. **(F,G)** Newly formed intermediate cell wall (DIC) separates four cells (GFP) contained within the pollen envelope. **(H)** Additional cycles of mitosis create a multicellular structure. CS, cytoplasmic strand; CW, cell wall; N, nucleus; n, nucleolus; PA, pollen aperture. Bar = 20 μm.

The nucleus of type I pollen (54.3% overall) contained a single nucleolus (Figure 2A). Prior to mitosis, the nucleus migrated from a strictly peripheral to a more central position and began moving rapidly while increasing in size, with cytoplasmic strands radiating from the nuclear periphery (Figures 2B,C) (Supplementary movie 2). After symmetric first mitosis (Figure 2D), the two resulting daughter cells remained mitotically active. Despite numerous rounds of synchronized mitoses (Figures 2F,G), the pollen did temporarily not increase in size; during this period of time, the cytoplasmic proportion of the individual cells increased while the vacuoles accordingly dwindled in size (Figures 2A–H). This type of pollen did not show any detectable starch accumulation. Type I development represents the major pathway of pollen embryogenesis under the conditions used in this study.

Type II showed an early difference to type I development (2.9% overall; see Supplementary movie 3). Here the nucleus prior to mitosis did not move to a central position and remained opposite of the pollen aperture. Furthermore, the cytoplasm remained largely peripheral and did not form cytoplasmic strands upon mitosis (Supplementary Figures 2B–F). Despite the formation of two nuclei of equal size and shape, a cell wall remained absent as judged by Differential Interference Contrast (DIC) microscopy (Supplementary Figures 2B–G) an unopposed movement of the two daughter nuclei throughout the cytoplasm was observed (Supplementary Figures 2B,C). Later this pollen started to deposit starch from day 3 onwards (Supplementary Figure 2D) and shortly died as indicated by cell shrinkage and the loss of GFP signal in the nucleus (Supplementary Figures 2F–H).

**Figure 3:**
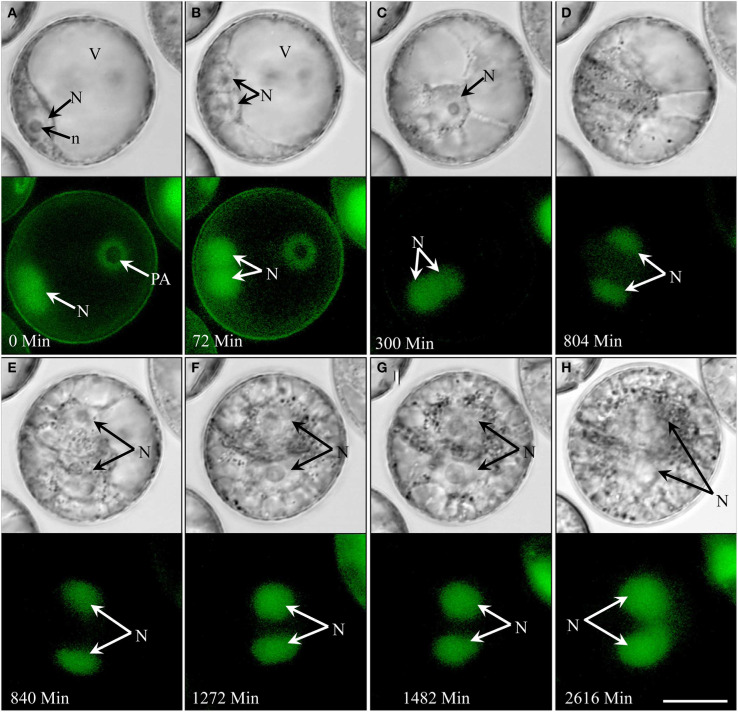
**Type III development over time (non-embryogenic pollen) shown by synchronously acquired DIC and fluorescence images**. After the initial symmetric division, the pollen remains bi-cellular, showing no further cell divisions but gradually accumulating starch while remaining viable throughout the observation period (14 days). **(A)** Uni-nucleate pollen with large vacuole and the nucleus residing opposite to the pollen aperture. **(B,C)** Symmetrical cell division. **(D,E)** Cell with two similar sized daughter nuclei and large vacuoles. **(F–H)** Increase of the cytoplasmic volume and starch accumulation. N, Nucleus; n, nucleolus; PA, pollen aperture; V, vacuole. Bar = 20 μm.

In type III development (4.3% overall; see Supplementary movie 4), the nucleus remained opposite of the pollen aperture at the time of symmetric cell division (Figures 3A,B). In contrast to type II development, the two daughter nuclei moved to the center of the cell (Figure 3C) with cytoplasmic strands radiating from their surface (Figure 3D), but showed no further mitotic activity. Type III pollen gradually accumulated starch without increasing in size over time (Figures 3D–H). In contrast to type II, type III pollen remained viable for more than 2 weeks, i.e., during the entire time of observation as shown by GFP signal in their nuclei (Figure 3H).

**Figure 4:**
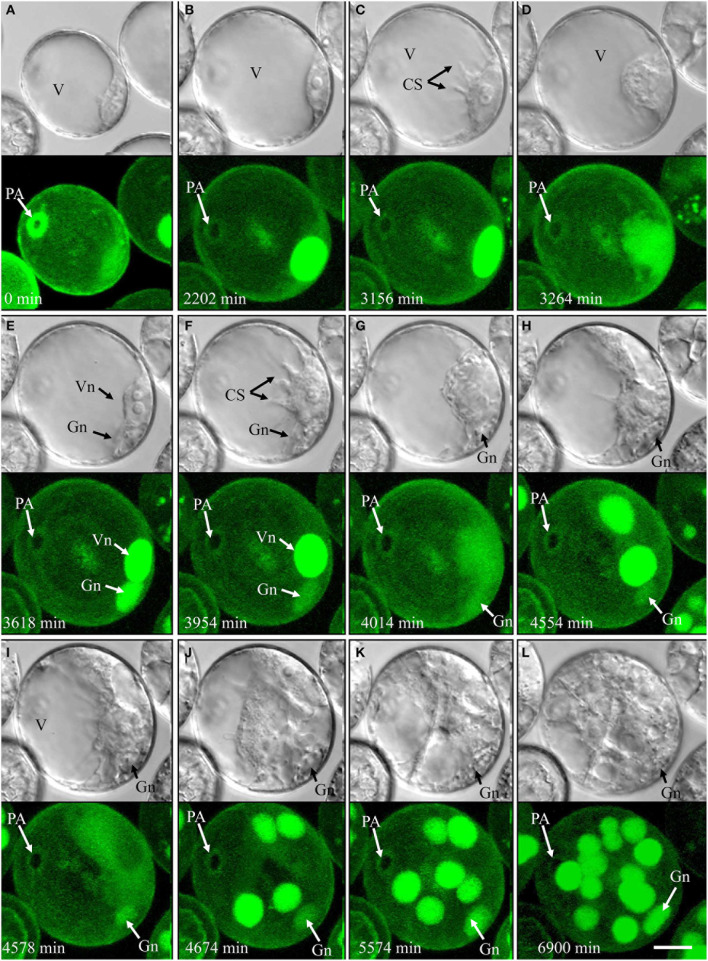
**Time-lapse of type IV development (embryogenic pollen) shown by synchronously acquired DIC and fluorescence images. (A)** Uni-nucleate pollen with large vacuole and thin layer of peripheral cytoplasm. **(B)** Uni-nucleate pollen increases in size. **(C)** Cytoplasmic strands appear prior to pollen mitosis I. **(D–F)** Large spherical vegetative-like nucleus and smaller ellipsoid generative-like nucleus after asymmetric division. **(G–L)** Synchronized mitotic events originate from the vegetative-like cell; note that the generative-like cell does not show any mitotic activity. CS, cytoplasmic strand; Gn, generative-like nucleus; PA, pollen aperture; V, vacuole; Vn, vegetative-like nucleus. Bar = 20 μm.

Of the pollen whose first mitosis was asymmetrical (types IV, V, and VI), only type IV followed an embryogenic pathway (4.3% overall; Figure 4, Supplementary movie 5). Type IV pollen significantly increased in size and cytoplasmic strands appeared prior to the first mitosis (Figure 4C). The nucleus, however, remained residing opposite of the pollen aperture (Figures 4A–D). The first mitosis was followed by an asymmetric cell division resulting in a large vegetative-like cell and a much smaller generative-like cell (Figure 4E). Whereas the latter remained inactive and opposite of the pollen aperture, the former showed active movements and the appearance of cytoplasmic strands that preceded a further mitosis (Figures 4F,G) resulting in two similar daughter nuclei (Figure 4H). Further synchronized divisions produced a multicellular structure that showed no sign of amyloplast formation and starch accumulation (Figures 4I–L). Compared to type I development, cell proliferation in type IV was delayed by several days. Over the time of observation, the generative-like cell degenerated in most cases; and although it remained viable in some cases, (Figures 4E–L).

Developmental type V (2.9% overall) was very similar to developmental type II in terms of cell size, thin-layered cytoplasm, starch accumulation and ultimate cell degeneration, except for the fact that here the first pollen mitosis was asymmetric.

**Figure 5:**
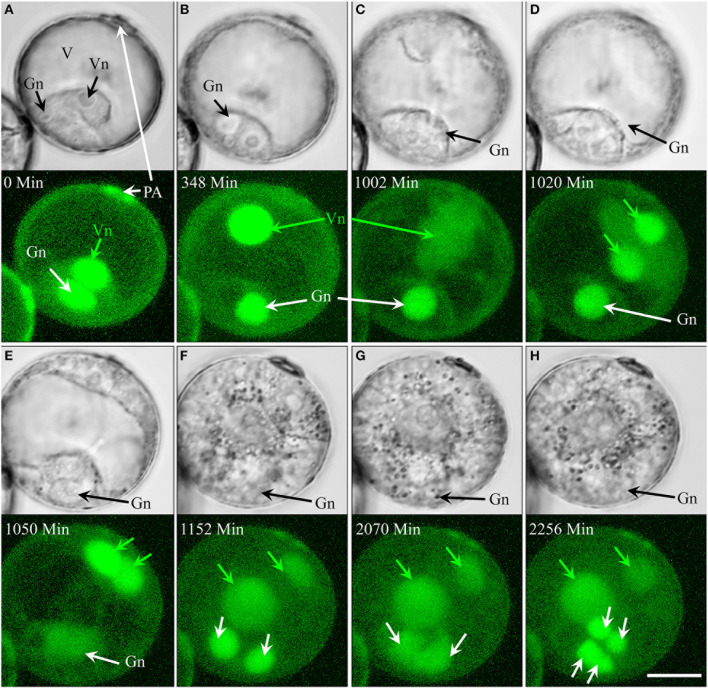
**Time-lapse of type VI (non-embryogenic pollen)**. Development shown by synchronously acquired DIC and fluorescence images of GFP. Green arrows indicate vegetative-like nucleus and its daughter nuclei. White arrows indicate generative-like nucleus and its daughter nuclei **(A,B)**. Asymmetric division resulted in a large vegetative-like and a small generative-like cell. **(C,D)** First mitosis of the vegetative nucleus; the spherical generative-like nucleus remains fixed opposite to the pollen aperture. **(E,F)** First mitosis of the generative-like cell. **(G,H)** Second symmetric and synchronized mitosis of the nuclei originated from the generative-like cell. Note that the nuclei derived from the generative-like nucleus are much smaller than those derived from the vegetative-like nucleus. Gn, generative-like nucleus; PA, pollen aperture; V, vacuole; Vn, vegetative-like nucleus. Bar = 20 μm.

Type VI pollen (7.1% overall; Supplementary movie 6) was characterized by forming a larger than usual, spherical, generative-like cell (Figures 5A,B) that was capable of undergoing two successive symmetric and synchronized divisions (Figures 5C–H). The nuclei produced by the mitotic activity of the generative-like cell remained within the original boundaries as defined after the first asymmetric division (Figures 5B–H). Of particular note is the small size of nuclei originating from the generative-like cell as compared to those derived from the vegetative-like cell (Figures 5F–H).

Of the pollen that failed to undergo mitosis, developmental type VII (1.4% overall; Supplementary movie 7) showed a remarkable increase in nuclear size over time (Supplementary Figures 3C–E). The pollen itself, however, did not expand and neither did form amyloplasts for starch accumulation. Cytoplasmic strands were not visible and the nucleus eventually disintegrated into multiple micronuclei (Supplementary Figures 3G–H). Despite fragmentation of the nucleus (on end of day 2 × of culture), the cell was still alive when observation ended.

Developmental type VIII (2.8% overall) pollen became exceptionally large (diameter 60 μm) but did not show any particular signs of development before dying within the first 2–3 days of culture.

Immature pollen of developmental type IX (20.0% overall) did not show any developmental changes during the time of observation; this pollen died sooner or later.

### Spontaneous genome doubling during pollen embryogenesis

**Figure 6:**
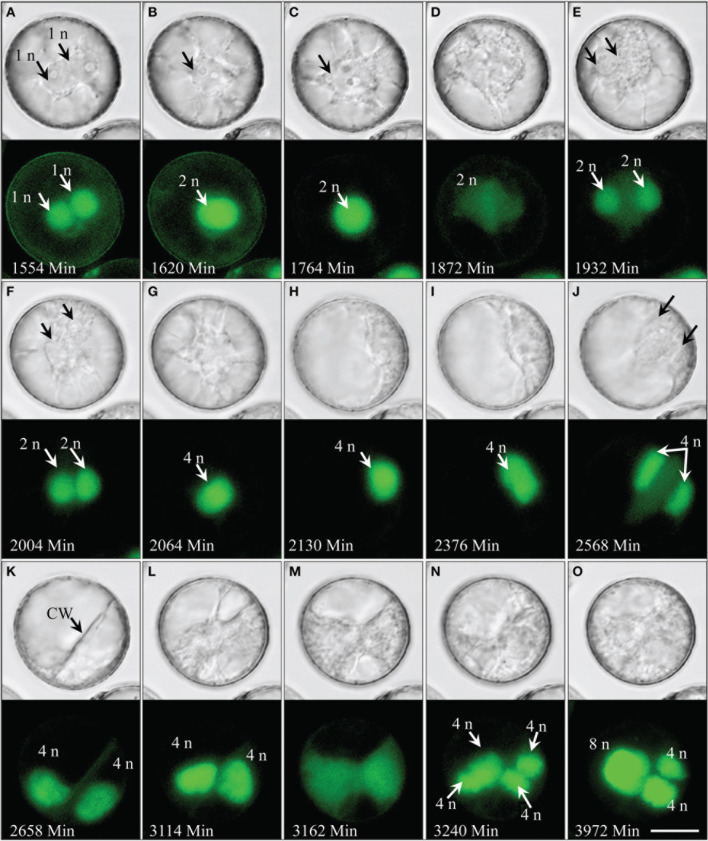
**Spontaneous genome doubling during pollen embryogenesis shown by synchronously acquired DIC and fluorescence images of GFP. (A)** Two haploid nuclei after the first pollen mitosis. **(B,C)** Nuclei adhere to one another and eventually fuse to form a diploid nucleus. **(D–F)** Second mitosis producing a pair of diploid sister nuclei. **(G–I)** The two diploid nuclei adhere to one another and fuse to a tetraploid nucleus. **(J,K)** Third mitosis resulted in two tetraploid nuclei. **(L–O)** Fourth synchronized mitosis resulting in four tetraploid nuclei, two of which later fuse to a single octaploid nucleus. CW, cell wall; n, haploid genome. Bar = 20 μm.

**Figure 7:**
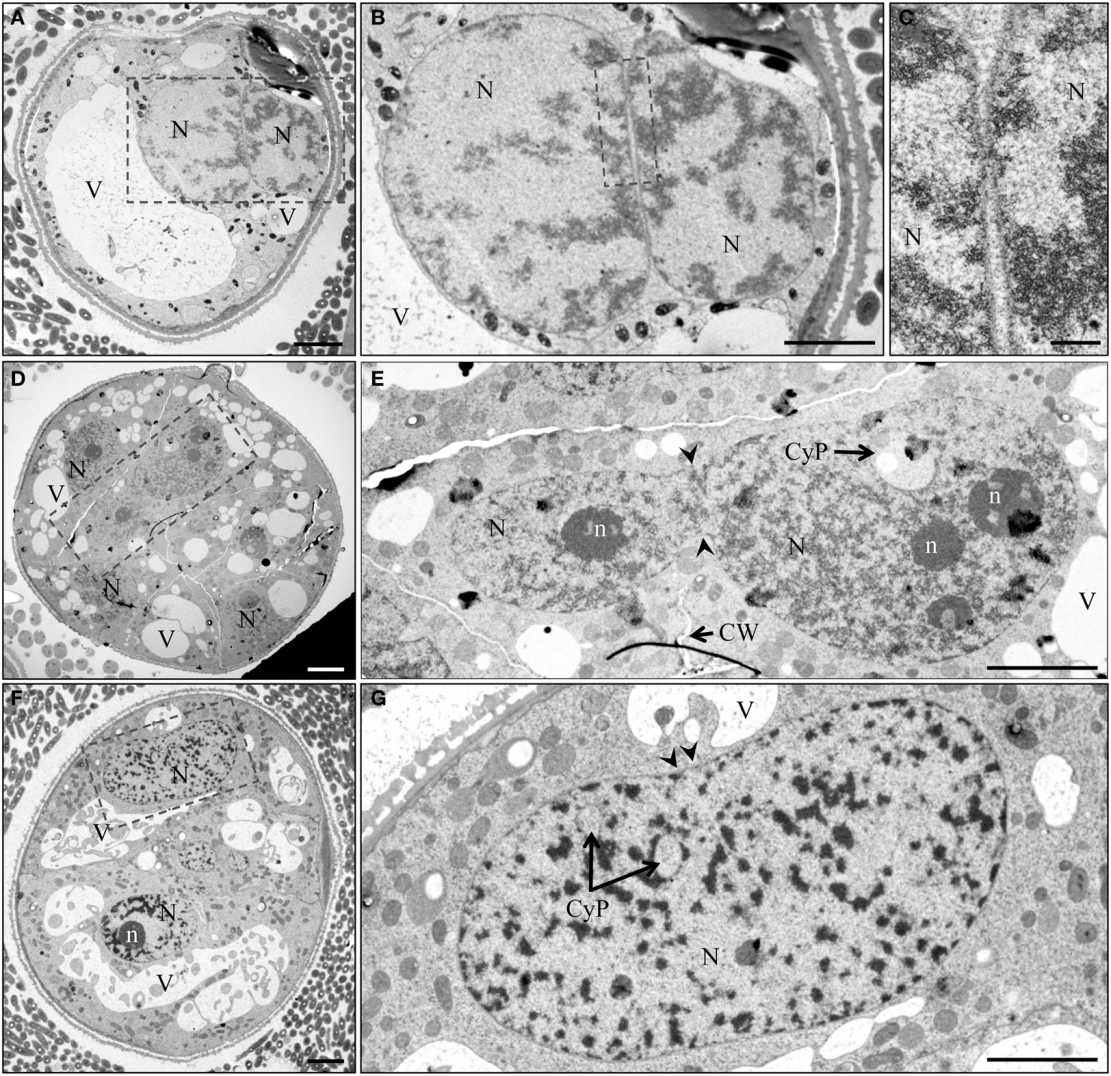
**TEM micrographs of nuclear fusion at different stages of pollen embryogenesis. (A)** Induced immature pollen during first day of culture with two nuclei in close vicinity. **(B,C)** Detail of **(A)** shows the absence of cell wall and the close proximity of the nuclear envelopes. Arrow indicates region of assumed membrane fusion. **(D)** Multicellular structure 7 days after initiation of pollen embryogenesis. **(E)** Two nuclei after fusion with incomplete cell wall formation near the site of nuclear fusion. **(F)** Multicellular structure 7 days after initiation of pollen embryogenesis. **(G)** Elongated nucleus with clear median constriction (arrowheads), cytoplasmic pockets and a narrow median band marking the site of fusion. CyP, cytoplasmic pocket; CW, cell wall; N, nucleus; n, nucleolus; V, vacuole. Bar = 20 μm.

**Figure 8:**
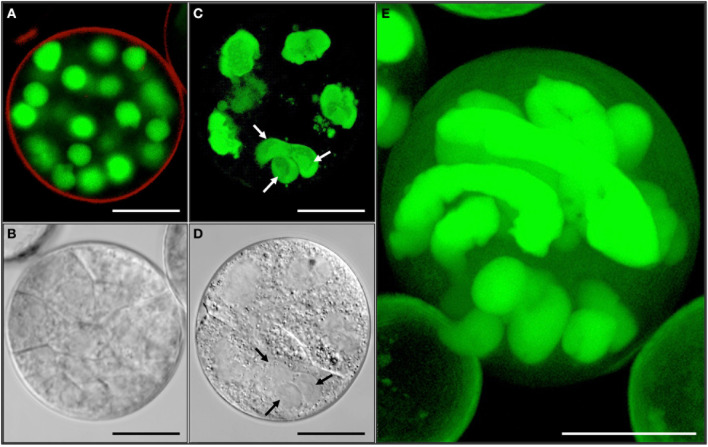
**Variable ploidy level in a multicellular structure shown by synchronously acquired DIC and fluorescence images. (A,C,E)** GFP. **(B,D)** DIC. **(A,B)** Haploid multicellular structure with cell walls and spherical nuclei. **(C,D)** Chimeric polyploid multicellular structure with irregular shaped nuclei often not separated by cell wall. Note the difference of nuclear sizes. Arrows refer to a possible triple fusion. **(E)** Multicellular structure with highly polyploid nuclei next to small spherical, likely haploid, nuclei. Bar = **A,B,D–E** = 10 μm; **C** = 1 μm.

Live-cell imaging revealed that nuclear fusion is a common process observed in more than 40% of the multicellular pollen and occurring throughout pollen embryogenesis (Figures 6–**8**; Supplementary movie 8) rather than being limited to a certain stage. Notably, polyploid products of multiple consecutive nuclear fusions were also frequently observed (Figures 6B,G,O, **8**). In some extreme cases, such processes resulted in large tube-shaped nuclei (Figure 8E).

More detailed information on the nuclear fusion process was obtained from electron microscopy studies. Nuclear fusion starts with a close alignment of two nuclei (Figures 7A, 8A–C), followed by fusion of the nuclear envelopes (Figures 7B,C). In some cases, cell wall was partially present at the site of the assumed nuclear fusion (Figures 7D,E). Nuclear fusion may account for the often occurring irregular shape of nuclei and the recurrent presence of cytoplasmic pockets within the nucleoplasm (Figures 7E,G). An intriguing observation was that of an elongated nucleus featuring a median invagination and unusual distribution of heterochromatin, which appeared to be absent from a narrow median band in the plane of the median constriction (Figure 7G).

The information gained from live-cell imaging and electron microscopy studies suggests that mitosis is not always followed by cell wall formation (Figures 6–8). Failure of cell wall formation can occur at any stage of pollen embryogenesis and so can nuclear fusion. This would explain the chimeric ploidy level often observed within individual multicellular structures (Figure 8E).

## Discussion

Only a few previous studies have followed the embryogenic development of isolated pollen in culture, e.g., Indrianto et al. (2001) in wheat as well as Kumlehn and Lörz (1999) and Maraschin et al. (2005) in barley. In these pioneering observations, time intervals of several hours or days were used, thus allowing only for a sketchy outline of the process. We here present a detailed monitoring of pollen embryogenesis from the vacuolated immature uni-nucleate barley pollen (the pre-mitotic microspore) until the formation of growing multicellular structures using a temporal resolution of only 3 min. The use of transgenic barley expressing a *GFP*-construct with nuclear-localization signal further greatly improved the accuracy of observations.

### Pollen development *in vitro*

Populations of immature pollen *in vitro* always show variability in the developmental state of individuals. Reasons for this heterogeneity include that pollen is typically isolated using a number of different spikes at once, that the florets of a spike show a developmental gradient along the rachis, and that nutrient provision and signal perception even of each individual pollen grain may well depend on the particular position within the anther. In addition, pollen of hybrid plants (that are typically used to produce doubled haploids in breeding practice) or of outbreeding species are genetically diverse due to meiotic segregation. In our experiments, 58.6% of pollen analyzed eventually formed multicellular structures (Table 1). This agrees with observations by Maraschin et al. (2005) who identified only three developmental pathways; whereas the present study enabled us to distinguish nine different types of pollen response to the given culture conditions (Table 1).

Indrianto et al. (2001) in wheat and Maraschin et al. (2005) in barley showed that multicellular structures were only obtained from immature pollen that were enlarged after induction treatment. In the present study, however, the type I pollen grains that underwent cell proliferation most efficiently did temporarily not increase in size during the initiation of cell proliferation. A very similar scenario has been shown in cultures of isolated wheat zygotes (Kumlehn et al., 1999; Figure 2I), where an initial series of cell divisions result in a step-wise decrease in cell size without substantial enlargement of the whole proembryo prior to the onset of its exponential growth based upon proliferation of small, cytoplasmic rich cells that are characteristic for the proper of globular zygotic embryos. Likewise, the formation and ongoing proliferation of small cells is indicative of the embryogenic nature of type I pollen. Type IV pollen did temporarily increase in size, which was associated with a remarkable delay in first mitosis. The resultant vegetative-like cell then behaved like the microspore cell of type I pollen in undergoing embryogenic development involving a successive decrease in cell size over an initial series of mitoses followed by continued proliferation of small cells. By contrast, other pollen grains that increased in size shortly after inductive treatment (types II, V, and VIII) eventually accumulated starch in amyloplasts and did not show continuous cell proliferation. Since amyloplasts are not unambiguously recognized by differential interference contrast microscopy used in this study, we have investigated samples of comparable developmental stages of barley pollen embryogenesis also by transmission electron microscopy and thereby proved that these structures are indeed starch-containing granules (data not shown).

Successful induction of embryogenesis in uni-nucleate pollen was usually associated with a first symmetric mitosis (92.7% of embryogenic pollen; type I) and only rarely with a first asymmetric mitosis (7.3% of embryogenic pollen; type IV). In contrast to pollen response type IV, both microspore daughter cells contributed to the cell proliferation following a first symmetric pollen mitosis (type I).

**Figure 9:**
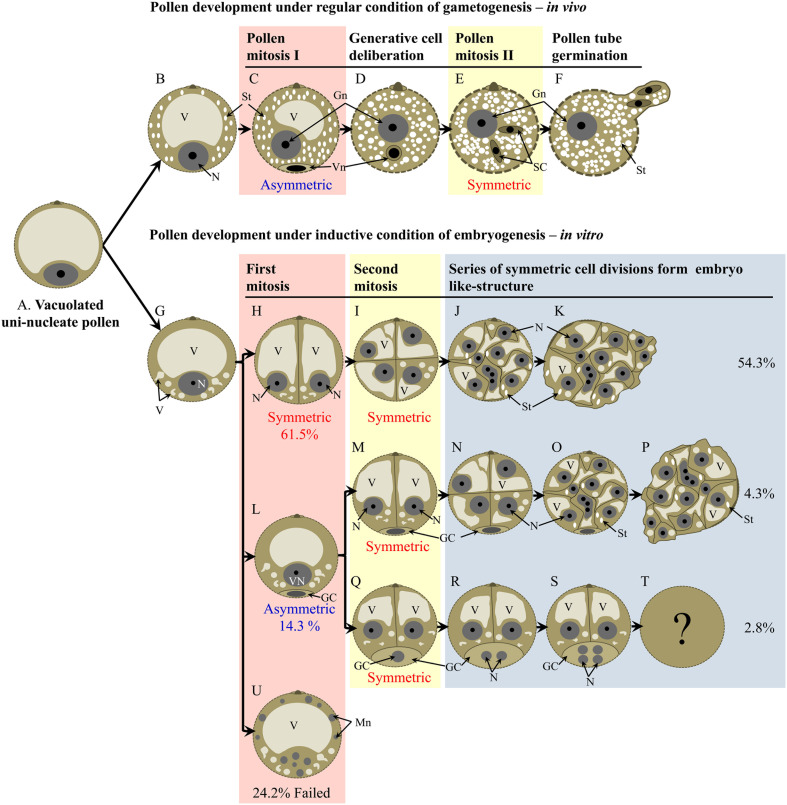
**Schematic model of main developmental routes of pollen during regular maturation and pollen embryogenesis**. **(A)** premitotic microspore, **(B)** commencement of starch accumulation in premitotic microspore, **(C)** product of asymmetric pollen mitosis I, **(D)** bicellular pollen with the generative cell deliberated from the cell periphery, **(E)** formation of two sperm via pollen mitosis II, **(F)** germination of mature pollen, **(G)** premitotic microspore with emerging small vacuoles, **(H)** bicellular pollen after symmetric 1st pollen mitosis, **(I)** 4-celled pollen after two rounds of symmetric cell divisions, **(J)** multi-cellular pollen, **(K)** pollen releasing proliferating tissue, **(L)** bicellular pollen after asymmetric 1st pollen mitosis, **(M)** tri-cellular pollen after symmetric division of the vegetative-like cell, **(N)** 5-celled pollen including a non-divided generative-like cell and 4 cells derived from the vegetative-like cell, **(O)** multicellular pollen including a non-divided generative-like cell and multiple cells derived from the vegetative-like cell, **(P)** multicellular pollen releasing proliferating tissue, **(Q)** tri-cellular pollen after symmetric division of the vegetative-like cell, **(R)** 4-nucleate pollen after division of the generative-like nucleus, **(S)** 6-nucleate pollen including 4 nuclei derived from the generative-like nucleus, **(T)** no information is available about pollen fate after stage S, **(U)** failure of cell division in microspore is associated with formation of micronuclei. GC, generative/generative-like cell; Gn, generative/generative-like nucleus; Mn, micro nucleus; N, nucleus; SC, sperm cell; St, starch; V, vacuole; Vn, vegetative/vegetative-like nucleus.

A comparison between regular pollen maturation and pollen embryogenesis is shown in Figure 9. The developmental pathways identified show three main routes based on the type of the first pollen mitosis (Figures 9H,L,U). In the most common route, first mitosis was symmetric and the two daughter cells proliferated synchronously to eventually form an embryo-like structure (Figures 9H–K). Starch was not detected in early stages, though some amyloplasts appeared later on (Figures 9J,K). In the less common second route, pollen firstly divided asymmetrically to produce generative and vegetative-like cells (Figure 9L). Depending on the fate of the generative-like cell, two sub-routes were identified. In the first sub-route, only the vegetative-like cell proliferated (Figures 9M–O) while the generative-like cell remained opposite to the cell aperture and often degenerated within the period of observation (Figures 9M–P). In the second sub-route both generative and vegetative-like cells underwent mitosis (Figures 9Q–S). In the third route, pollen failed to undergo first mitosis and in some case the nucleus was degenerated into small micronuclei (Figure 9U).

### Nuclear fusion leads to genome doubling

Spontaneous genome doubling during pollen embryogenesis can produce doubled haploid plants that, in contrast to haploids, show normal fertility.

Sunderland et al. (1974) proposed the fusion of mitotic nuclei as an explanation for genome duplication in embryogenic pollen grains of *Datura*. An alternative mechanism was published by Seguí-Simarro and Nuez (2008) who observed that karyokinesis is followed by a disrupted cytokinesis, which allows the daughter nuclei to fuse within the same cytoplasm. Lee and Chen (1987) and Kasha (2005) claimed the fusion of generative and vegetative nuclei in cultures of barley pollen after the degradation or incomplete assembly of the separating cell wall.

Our observations revealed that mitosis is not always followed by cytokinesis which allows mitotic daughter nuclei to fuse. Indeed, nuclear fusion turned out to be the only means of genome doubling in the cultures analyzed in the present study. It could occur at any time during pollen embryogenesis, which explains the chimeric ploidy status of individual multicellular structures (Figures 6, 8) and plants. González-Melendi et al. (2005) showed that when nuclei coexist within the same cytoplasm, their envelopes may fuse. However, the same authors also argued that the absence of cell wall is not sufficient to explain nuclear fusion. There are indeed many examples of stable multinucleate cells that occur naturally, e.g., bi-nucleate tapetal cells, coenocytic endosperm and the female gametophyte, or can be experimentally-induced (Risueño et al., 1968; Nishihama et al., 2001; Park and Twell, 2001; Olsen, 2004). Consequently, hitherto unknown factors must exist, that stimulate attachment and fusion of nuclear envelopes (Chen et al., 1984; González-Melendi et al., 2005; Seguí-Simarro and Nuez, 2007).

Fusion between vegetative and generative nuclei was never observed in the present study. Every asymmetric pollen mitosis I ended with a physical barrier between vegetative and generative-like cells, which effectively precluded their respective nuclei from fusing. The integrity of the separating cell wall is also supported by the observation that in cases where generative-like cells were able to undergo symmetric divisions, all daughter cells remained within the boundary of the original generative-like cell (Figure 5).

The high frequency of whole genome duplication events and resultant polyploid nuclei observed is not associated with a corresponding proportion of high polyploidy amongst the regenerants, as was shown for example in the present study by the flow-cytometric analysis of the *gfp*-transgenic plants generated via agroinoculation of embryogenic pollen cultures. It is well conceivable that a ploidy larger than twice the normal somatic value can lead to disadvantages in further embryogenic development and regeneration which effects selection in favor of viable individuals in the ploidy range between 1 and 4 n with a further preference of diploids. Also in the ploidy-chimeric structures frequently found in this study, the same selection principle may effect a preferential development of cells and tissue domains having a ploidy within this tolerable range.

### Summary and perspective

Pollen embryogenesis can be followed after symmetric or asymmetric mitosis. The appearance of starch granules or pollen expansion prior to or right after pollen mitosis I is associated with failure of pollen embryogenesis under the conditions used in this study. Under the culture conditions used, nuclear fusion was the only mechanism of genome doubling and could occur at any developmental stage during pollen embryogenesis, provided cell wall formation had failed entirely or locally. In the rare occasion of a generative-like cell showing mitotic activity, the nuclei remained significantly smaller than those of normal embryonic cells. This makes the contribution of these cells to embryogenesis highly doubtful. Because cultures of immature pollen are highly heterogenic, it was necessary to follow the fate of individual pollen, in order to unambiguously identify and validate those developmental types that truly account for pollen embryogenesis.

The descriptive information provided here will be a valuable source for the evaluation of pollen cultures used to produce doubled haploid plants. This will especially apply for the establishment and improvement of protocols for recalcitrant species (e.g., rye and oats) or genotypes that have so far been hardly amenable to pollen embryogenesis. The thorough characterization of two embryogenic pathways and their unambiguous discrimination from seven non-embryogenic types of response as was performed in the present study we also consider as a vital prerequisite for future transcriptomics and metabolomics approaches relying on the collection of individually selected pollen, which may help to cope with the unavoidable heterogeneity in pollen populations. Also, the findings presented here on the mechanism and temporal occurrence of whole genome duplication events are likely to have implications on the utilization of embryogenic pollen cultures in induced mutagenesis, genetic transformation and genome engineering approaches with regards to zygosity and chimerism of the genetic alterations obtained in doubled haploids. In addition, the novel technical opportunities provided by the experimental setup established and utilized in the present study may facilitate the elucidation of the still unknown molecular triggers of pollen embryogenesis by over-time observation of fluorescence-tagged subcellular structures or candidate proteins essentially involved in this process.

## Methods

### Genetic transformation of barley using embryogenic pollen cultures

Barley transformation of the winter type cv. Igri using hygromycin as selective agent in the culture media was performed as previously described (Kumlehn et al., 2006). The hypervirulent *A. tumefaciens* strain LBA4404/pSB1 (Komari et al., 1996) carrying the binary vector pGH252n was used to inoculate embryogenic pollen. The binary vector was cloned followed standard procedures described in detail in the Supplementary materials and methods section, and its introduction into agrobacteria was performed by electroporation.

### Molecular analysis of transgenic plants

Genomic DNA prepared from leaf material (Pallotta et al., 2000) was analyzed by standard PCR using primers for the coding sequence of the *HPT* gene GH-HYG-F1 (5′-GATCGGACGATTGCGTCGCA-3′) and GH-HYG-R2 (5′-TATCGGCACTTTGCATCGGC-3′), or *GFP* GH-GFP-F1 (5′-GGTCACGAACTCCAGCAGGA-3′) and GH-GFP-R2 (5′-TACGGCAAGCTGACCCTGAA-3′). For DNA gel blot analysis, genomic DNA was digested with *Hind*III, separated in 0.8% (w/v) agarose gel (30 μg per lane) and blotted onto Hybond N+ membrane (Amersham, Braunschweig, Germany) by capillary transfer under alkaline conditions according to the manufacturer's instructions. Membranes were hybridized with DIG-labeled *HPT* according to the manufacturer's instructions (Roche, Mannheim, Germany). Hybridization and detection was performed according to the protocol for non-radioactive DNA gel blot experiments (Roche, Mannheim, Germany).

### Analysis of reporter gene expression using confocal laser scanning microscopy

Leaves, roots and pollen from T_0_ transgenic plants were analyzed for the presence of GFP with a Zeiss LSM 510 META CLSM (Carl Zeiss Microscopy GmbH, Jena, Germany) using a 488 nm laser line for excitation. GFP signals were detected with a 505–530 nm bandpass filter. Auto-fluorescence of chlorophyll was detected with a 650 nm long-pass filter.

### Ploidy analysis and colchicine treatment

Ploidy level of regenerants was assessed using a Ploidy Analyser PA I (Partec GmbH, Münster, Germany). Nuclei were stained with CyStain UV (Partec GmbH, Münster, Germany) according to the manufacturer's instructions. Haploid regenerants were treated with colchicine to induce genome doubling (Takamura and Miyajima, 1996). A detailed description is provided in the Supplementary materials and methods section.

### Live-cell imaging

Live-cell imaging was performed as described by Daghma (2011). Cultures of transgenic immature pollen were observed over a period of up to 15 days. Pollen was subjected to inductive treatment involving incubation of dissected anthers in 0.4 M mannitol at 25°C for 1 day followed by 4°C for 1 day before isolation and culturing in SMB medium for 1 day at 25°C in the dark. The capture of images was started immediately after pollen had been transferred into KBP medium. GFP was excited with a 488 nm argon-krypton laser line. DIC images were acquired with a HeNe 633 laser line. Z-stacks of 9 images were taken every three min with a spacing of 1.5–4 μm depending on expansion of pollen during the development. Scanning of every Z-stack took 80 s. To reduce the risk of bleaching, laser intensity was kept below 4% emission. The developmental progress of a total of 71 individual immature pollen grains was analyzed in five separate experiments.

### Transmission electron microscopy

For transmission electron microscopy, pollen was prepared using high pressure freezing and freeze substitution as described by Daghma et al. (2011).

## Author contributions

Diaa Eldin S. Daghma, Jochen Kumlehn, and Michael Melzer designed the research. Diaa Eldin S. Daghma and Goetz Hensel performed the experiments and analyzed the data. Twan Rutten contributed to time-lapse imaging. Diaa Eldin S. Daghma, Goetz Hensel, and Jochen Kumlehn wrote the manuscript.

### Conflict of interest statement

The authors declare that the research was conducted in the absence of any commercial or financial relationships that could be construed as a potential conflict of interest.
