# Supplementary material for: Cellular dynamics during early barley pollen embryogenesis revealed by time-lapse imaging
Source: Front Plant Sci. 2014 Dec 8;5:675. doi: 10.3389/fpls.2014.00675 (PMC4259004; doi:10.3389/fpls.2014.00675)
Supplement: Supplementary file 9 [file DataSheet1.DOCX]

***Supplementary Material***

**Cellular dynamics during early barley pollen embryogenesis revealed by time-lapse imaging**

**Diaa Eldin S. Daghma^1,2^, Goetz Hensel^1^, Twan Rutten^1^, Michael Melzer ^1^ & Jochen Kumlehn^1*^**

**^1^** Department of Physiology and Cell Biology, Leibniz Institute of Plant Genetics and Crop Plant Research, Gatersleben, Germany

^2^ Department of National Gene Bank and Genetic Resources, Agriculture Research Center, Giza, Egypt

***Correspondence:** Dr. Jochen Kumlehn, IPK-Gatersleben Institute, Department of Physiology and Cell Biology, Laboratory of Reproductive Cell Biology, Correnstr 3,
Gatersleben, 06466, Germany, [kumlehn@ipk-gatersleben.de](mailto:Kumlehn@ipk-gatersleben.de)

**1. Supplementary methods**

- 1. **Agrobacterium strain and plasmids**

The hypervirulent *A. tumefaciens* strain LBA4404/pSB1 (Komari et al., 1996) carrying the binary vector pGH252n was used to generate transgenic barley plants (Fig. 1). The *Sal*I/*Eco*RI fragment of pC1300intA-NLS-sGFP (kindly provided by P. Owerkerk, Leiden, Netherlands) containing the NLS of *SV40* (van der Krol and Chua, 1991) and an enhanced version of the *GFP* gene (Chiu et al., 1996) was cloned into the *Sal*I/*Not*I digested vector pd35S-Nos-ABM (Himmelbach et al., 2007). Both fragments were treated with Klenow enzyme for a fill-in reaction prior to the ligation that resulted in pd35S-NLS-sGFP. *Acc*65I/*Hind*III fragment containing the *NLS:sGFP* and the *NOS* terminator was introduced into the corresponding sites of plasmid pUbi-AB-M (DNA Cloning Service, Hamburg, Germany) harbouring the maize *UBIQUITIN1* promoter with first intron (Christensen and Quail, 1996) yielding vector pGH132. The *Sfi*I fragment of vector pGH132 harbouring the full *GFP* expression cassette was introduced into the appropriate sites of plasmid p6U (DNA Cloning Service, Hamburg, Germany) to generate vector pGH252n (Fig. 1).

- 1. **Colchicine-induced genome doubling**

Plants with at least 2 tillers were removed from the pots and the roots were carefully washed with tap water, cut back to 1 cm, immersed in aqueous 0.1% (v/v) colchicine solution supplemented with 0.8% (v/v) dimethyl sulfoxide (DMSO) and 0.05% (v/v) Tween-20, and incubated at room temperature in dark for 5 h. Then, the roots were carefully rinsed with tap water for a few minutes and the plants replanted in soil. After re-establishment, plants were vernalized in a cold room at 2^o^C with 8 h day length for 6 weeks. Grains of doubled haploids were harvested at full maturity.

1. **Supplementary figures**


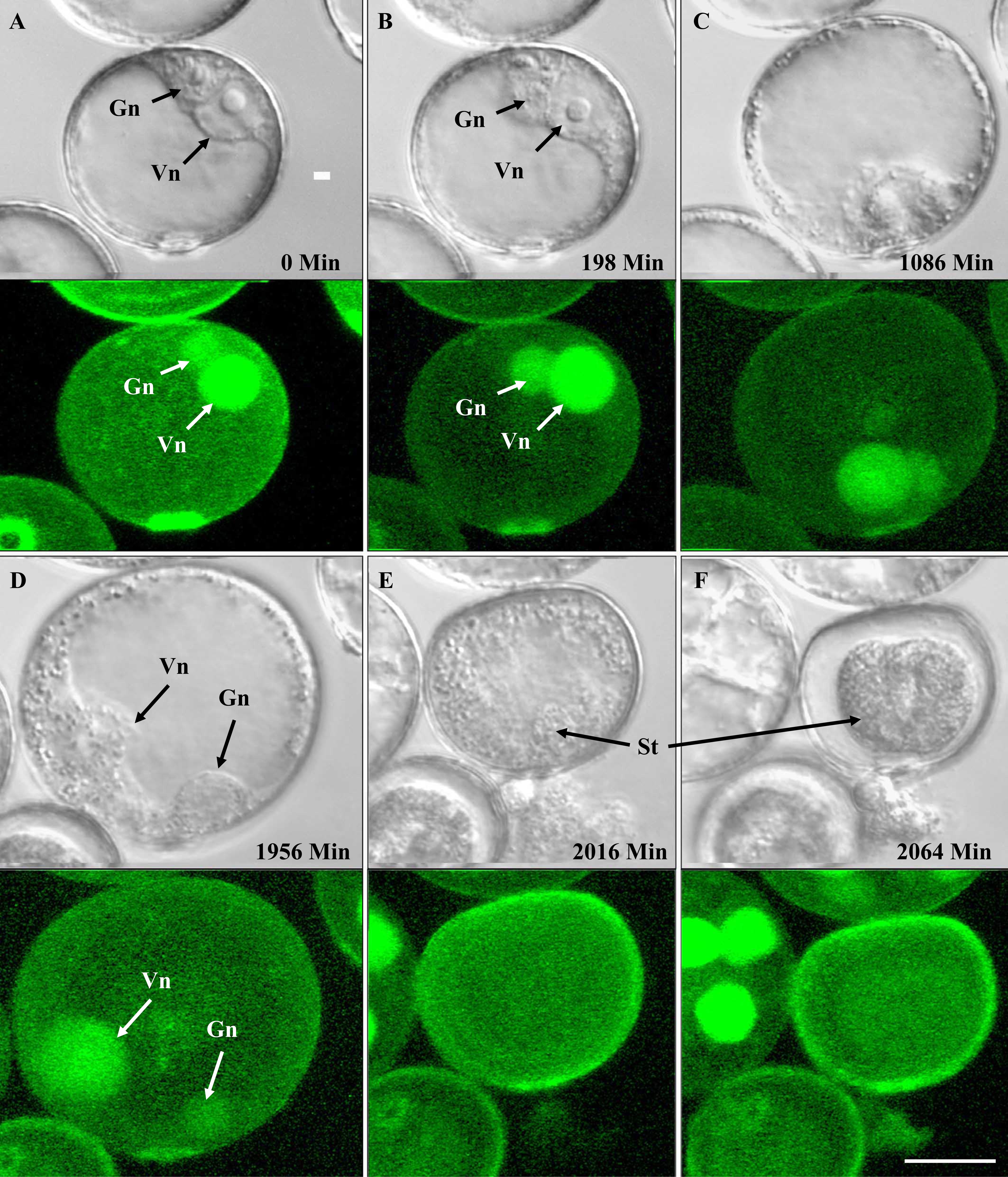


Supplementary figure 1. Time-lapse of bi-cellular pollen shown by synchronously recorded DIC and fluorescence images of GFP expression. (A) Bi-cellular pollen with large vacuole, a large vegetative and a smaller spherical generative nucleus. (B, C) Pollen size increases and starch granules accumulate while the nuclei move towards the vicinity of the pollen aperture. (D) Vegetative nucleus detaches from the generative cell. (E, F) Cell death indicated by the disappearance of GFP and a general cellular collapse. Gn, generative nucleus, St, starch granules; Vn, vegetative nucleus. Bar = 20 µm.


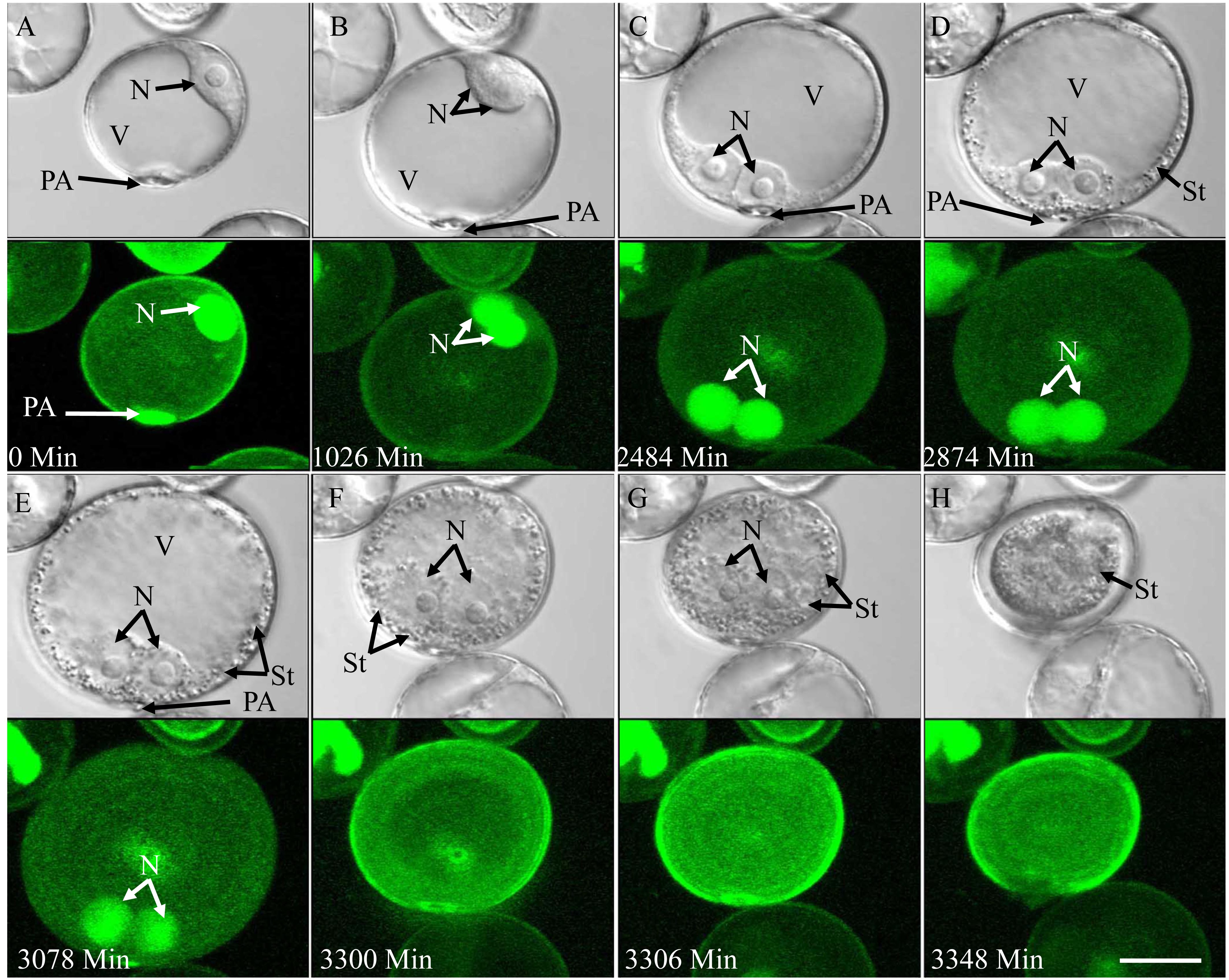
 Supplementary figure 2. Time lapse of type II development (non-embryogenic pollen) shown by synchronously recorded DIC and fluorescence images of GFP expression. (A) Uni-nucleate pollen with large vacuole and nucleus, containing a single nucleolus, opposite to the pollen aperture. (B, C) Size of pollen increases during the first mitosis; note the absence of cytoplasmic strands (DIC). (D, E) Daughter nuclei in close vicinity (GFP) without discernible cell wall (DIC). (F-H) The two nuclei remain side by side while they translocate to the site of the pollen aperture; cytoplasm remains peripheral and there is no evidence for a cell wall formation. (D-H) Day three onwards: the cell accumulates starch. (F-H) Cell death indicated by a decrease in cell size and disappearance of the GFP fluorescence. N, nucleus; PA, pollen aperture; St, starch; V, vacuole. Bar = 20 μm


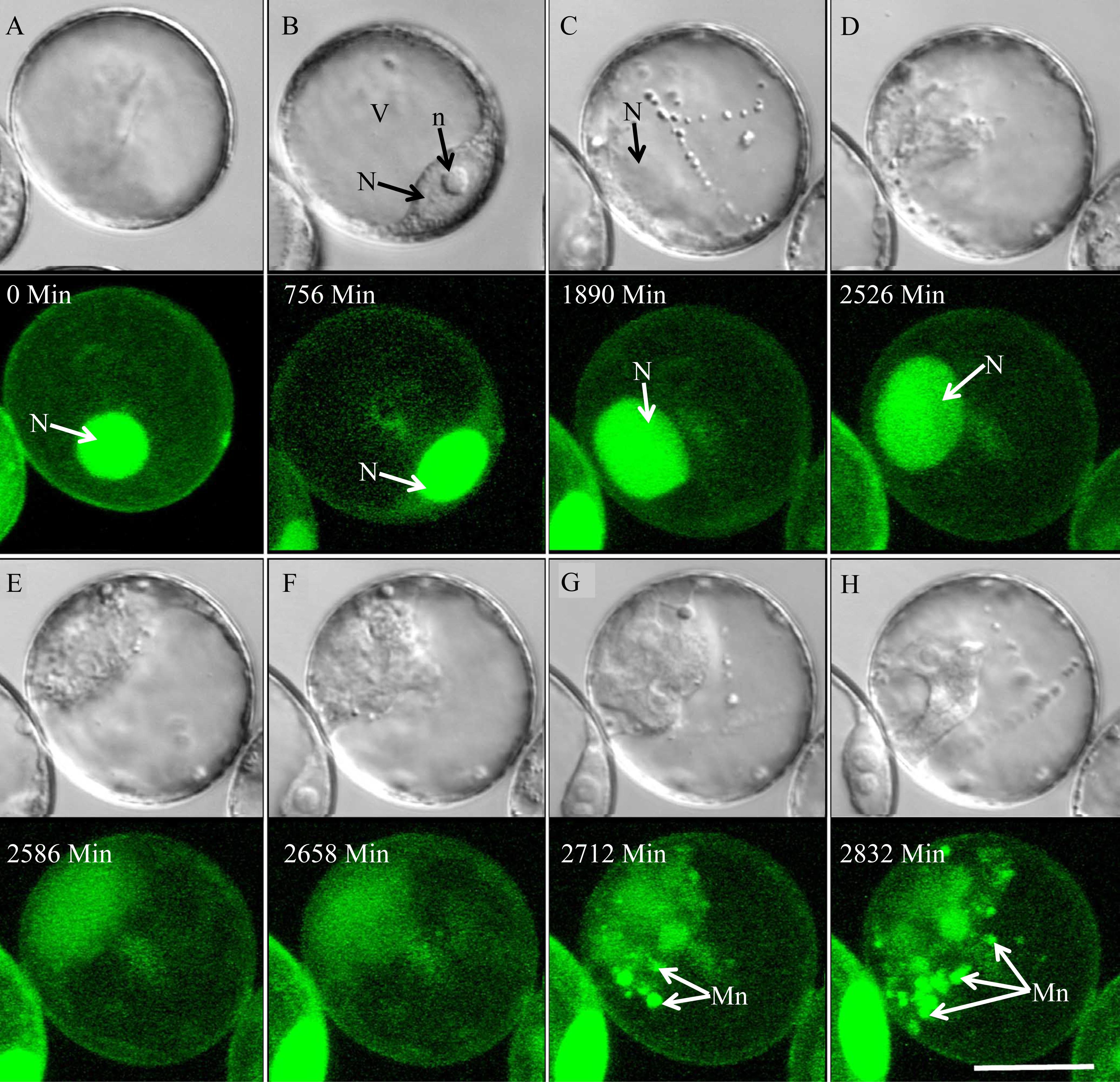
 Supplementary figure. Time lapse of type VII development (non-embryogenic pollen) shown by synchronously recorded DIC and fluorescence images of GFP expression. The pollen nucleus fails to divide and disintegrates into multiple micronuclei. (A, B) Highly vacuolated uni-nucleate pollen (microspore). (C, D) Gradual increase in nuclear size. (E, F) Blurred GFP signal of the nucleus. (G, H) Fragmentation of the nucleus into several micronuclei as indicated by separpate GFP signals. Mn, micronuclei; N, nucleus; n, nucleolus; V, Vacuole. Bar = 20 µm.

1. **Supplementary movies**

**Supplementary** **movie 1.** Bi-cellular pollen shown by synchronously acquired DIC and fluorescence images.

**Supplementary** **movie 2.** Type I development (embryogenic pollen) shown by synchronously acquired DIC and fluorescence images.

**Supplementary** **movie 3.** Type II development (non-embryogenic pollen) shown by synchronously acquired DIC and fluorescence images.

**Supplementary** **movie 4.** Type III development (non-embryogenic pollen) shown by synchronously acquired DIC and fluorescence images.

**Supplementary** **movie 5.** Type IV development (embryogenic pollen) shown by synchronously acquired DIC and fluorescence images.

**Supplementary** **movie 6.** Type VI development shown by synchronously acquired DIC and fluorescence images.

**Supplementary** **movie 7.** Type VII development (non-embryogenic pollen) shown by synchronously acquired DIC and fluorescence images.

**Supplementary** **movie 8.** Spontaneous genome doubling during pollen embryogenesis shown by synchronously acquired DIC and fluorescence images
